# Nurses’ activities and time management during home healthcare visits

**DOI:** 10.1111/scs.12813

**Published:** 2019-12-21

**Authors:** Heli Vaartio‐Rajalin, Yvonne Näsman, Lisbeth Fagerström

**Affiliations:** ^1^ Faculty of Pedagogy and Welfare Studies Åbo Akademi University Vasa Finland; ^2^ Nursing Program Novia University of Applied Sciences Åbo Finland; ^3^ Faculty of Health and Social Sciences University of South‐Eastern Norway Kongsberg Norway

**Keywords:** home care, nursing activities, observation, person‐centred care, time management

## Abstract

**Aim:**

To describe nurses’ activities and time management during HHC visits from the perspective of master’s‐level nursing students.

**Background:**

The shift from community‐based hospitals to home‐based, person‐centred services for patients with a variety of acute or chronic health problems challenges nurses’ professional competence and time management during home healthcare visits.

**Design and methods:**

A cross‐sectional study in accordance with STROBE guidelines. Observation sheets (n = 196) from two municipal home healthcare organisations were analysed with descriptive quantitative analysis.

**Ethical issues and approval:**

While no external ethical committee evaluation was necessary for this quality improvement study, research ethical principles were followed.

**Results:**

The nurses spent 50% of each eight‐hour shift on indirect patient contact activities and about 38% on direct patient contact activities. The majority of activities underlying the home visits could be linked to long‐term illnesses: medication (57%), blood samples (23%), wound care (17%) or measurement of blood pressure (14%). Patient education was offered during only 3.5% of visits.

**Limitations:**

The accuracy of the students’ observations is related to their individual capacity to objectively and selectively observe.

**Conclusions:**

There were a number of activities conducted *for the patient*, to promote continuous intra‐ and interprofessional patient care, but fewer nursing activities conducted *with the patient.* To ensure integrated, person‐centred, safe patient care, vital reforms are needed.

**Relevance to clinical practice:**

The appropriate balance between indirect and direct patient contact activities should be discussed intra‐ and interprofessionally, delineated and made explicit in nurses’ work plans and nursing documentation, alongside discussions pertaining to relevant resource allocation.

## Background

Recognising that patients prefer to be treated at home and that home‐based care can reduce care delivery costs system‐wide, politicians and leaders have begun to prioritise noninstitutional care settings 1 and intermediate care 2. This shift from community‐based hospitals to home‐based, person‐centred services for patients with a variety of acute or chronic health problems challenges nurses’ professional competence and time management during home healthcare (HHC) visits.

Traditionally, the aim of HHC has been to facilitate discharge from hospital and prolong individuals’ prospects of living at home, even for those with chronic conditions. HHC has generally been offered when other healthcare services have been deemed inconvenient or unsuitable for individuals with altered functional capacity or somatic illnesses. In Finland, HHC is comprised of physician‐ordered interventions and social services. This can include the taking of blood samples; monitoring of medicine compliance and/or patients’ clinical condition; or provision of functional support for activities of daily living. HHC in Finland can accordingly be considered integrated, highly interprofessional care. The majority of HHC patients have typically been older persons with primary care needs 3, 4. Still, during the three last decades, HHC has also been offered cumulatively to ill working‐age individuals, individuals with mental health problems, families with ill children and terminal‐phase patients.

As seen in a scoping review 5, HHC nurses today are responsible not only for the provision of primary healthcare interventions in the home environment but also for advanced care. Advanced care includes holistic assessment of patients’ physical and psychological status; the planning, coordination, implementation and evaluation of patient‐centred care; management of advanced medication; and provision of patient and family caregiver education. It also includes the management of new technical/digital developments that facilitate interprofessional coordination, rehabilitation and research assistant duties. Embedded into these responsibilities is even that HHC nurses act as a ‘communications hub’, facilitating interprofessional communication, which has been shown to reduce the re‐hospitalisation of high‐risk patients by 8–33 per cent 6. One can question whether it is possible for HHC nurses to accomplish all of the tasks mentioned above, especially because the number of HHC patients has increased but the number of HHC staff has not 7, 8. This disparity in numbers has resulted in increased pressure on HHC nurses’ time management skills 9 and discussions about HHC staff’s educational requirements and how to improve HHC staff motivation 7, 8, 10.

The home as a context for care not only can promote patients’ autonomy and well‐being, but also can increase safety risks 5. The HHC setting provides healthcare staff with an optimal opportunity to identify and respond to patients’ and their families’ needs, which can be perceived as the provision of patient‐ or person‐centred care. Person‐centred care can be defined as care that is respectful of and responsive to individual patient preferences, needs and values, where all clinical decisions are guided by the views of the patient 11. Person‐centred care also includes respect for the personal narratives that reflect a person’s sense of self, lived experiences and relationships, that is personal knowledge, and the safeguarding of partnership in the caring relationship through shared decision‐making and meaningful activities in a personalised environment 12, 13, 14.

A patient‐centred approach is one of the key components of high‐quality integrated care. Integrated care has been linked to increased service efficiency, decreased costs (60–70% reduction in emergency room admissions, 50% reduction in hospital admissions; Ref. 15, improved equity in service uptake, better health literacy and self‐care, increased satisfaction with care, and improved patient–healthcare provider relationships 16. Yet in an analysis of care and service plans (n = 437) in HHC in Finland 17, researchers found that HHC care planning was often illness‐centred and focused on patients’ diseases and/or functional/cognitive disabilities, with the plans themselves heavily focused on classification and composed using passive expressions. Moreover, documentation was based on the philosophy of ‘doing for’ patients rather than ‘doing with’. Excepting components related to respiration, follow‐up treatment, life cycle and health behaviour, which were not investigated in that study, medication was the most reported component (93%), followed by self‐care (85%), coping (78%), physical activity (30%) and skin integrity (25%). The researchers also found that most care and service plans were designed emanating from care professionals’ point of view 17.

Care delivery reforms worldwide will benefit HHC 1, and it is expected that HHC will become a significant employer of Registered Nurses (RNs). Still, concerns have been raised about HHC staff turnover and clinical training, for example in skilled areas of care 18. HHC internships for nursing students are not be systematically included in nursing education in Europe 19, even though many nursing students work at HHC during school breaks. This may stem from assumptions that HHC work is easy and nonchallenging and entails providing primary care 20.

To guarantee a competent, motivated and enabled HHC workforce in the future, it is vital to point out the reality of HHC to nursing students, nurse educators, leaders and policymakers. While there is evidence that the highest‐staffed nursing homes provide better care 21, the relationship between number of staff and care quality is not linear 10. Very few studies on HHC nursing interventions have been conducted since the turn of the century, and none have included an assessment of time management in the HHC setting.

The aim of this study was to describe nurses’ activities and time management during HHC visits from the perspective of master’s‐level nursing students. Unlike other studies in which a focus is placed on nurse–patient ratios, staffing numbers or workload, here the focus lay on students’ one‐to‐one nonparticipatory observations of nurses’ work in HHC. The research question was as follows:

- What does an HHC nurse do for patients before, after and during home visits?

This study is a part of research project with the overall aim to develop quality criteria for competent, safe, effective and person‐centred advanced care at home.

## Methods

This was a cross‐sectional descriptive study at two municipal HHC organisations in Finland. Strengthening the Reporting of Observational studies in Epidemiology (STROBE) guidelines were followed throughout. In autumn 2017, students in a master’s‐level caring science programme (n = 18) received training in observation technique and, under the auspices of a healthcare administration course part of their degree programme, collected data at two municipal HHC organisations. The healthcare administration course that the students participated in encompassed the study of various classification systems and classification theories. The observation sheet for this study was based on a differentiation between activities involving indirect and direct patient contact 22, 23. Inspiration for this approach was taken from measurement instruments used in Finland to monitor nursing care intensity, which the students here also studied as part of their degree programme.

In this study, the students engaged in the one‐to‐one nonparticipatory observation of HHC nurses’ (n = 18) work, observing 2–3 work shifts per nurse. Nonparticipatory observation involves observing participants without active participation and is used to understand a phenomenon by accessing a setting while still maintaining detachment from the activities being observed 24. To document the HHC nurses’ activities, observation sheets were created (see Table 1).

**Table 1:** Structure of observation sheets

Indirect patient contact activities	Direct patient contact activities
Planning Planning home visit Ordering/managing material/medicines needed for visit Checking laboratory results	Nursing care Monitoring patients’ illness, symptoms, nutrition and fluid balance Hygiene and elimination Activity and mobility
Documentation Patients’ care plans Statistical lists (automobile/travel logbook)	Medication
Travel time From healthcare station to patient residence From patient residence to patient residence Return to healthcare station at the end of a work shift	Caring discussion
Telephone calls Patients Acute care Pharmacy Other consultant (e.g. other nurse or physician)	Other activities
Professional meetings With other nurses or physicians (to discuss patient matters without patients)	
Other activities Filling patients’ pill dispensers Helping colleagues with IT problems Work breaks Administrative workplace meetings (outside observers not allowed), Interviews for research project General correspondence (emails, etc.)	

The data collected included students’ observations of HHC nurses’ activities and time management and some demographic patient data (gender, living/housing arrangements, main diagnosis, reason for home visit). The observation data (n = 196 observation sheets) were synthetised using quantitative descriptive methods (frequency, percentage, mean, mode), but due to small subsample sizes, no correlations or statistical significance tests were calculated.

### Research ethics

The ethical principles delineated by the Finnish National Board on Research Integrity 25 were followed during the entire course of the study, including informant recruitment, data collection, data analysis and publication. No external ethical committee evaluation for the study was considered necessary, because the organisations overseeing the data collection procedures categorised it as the starting point for a quality improvement project.

The healthcare administration course part of the master’s‐level caring science programme was obligatory for those students seeking to minor in healthcare administration. Because the focus of the course was on healthcare administration research and development, those overseeing the degree programme sought a project whereby the students could gain practical research experience. This included students following a research process from start to finish: from plan (written by the research leader) to published results (written by the research team). The students’ were tasked with collecting data. While one can consider this project to be more of a didactical exploration than an ordinary research project, it nonetheless yielded useful information for the various stakeholders involved, that is the university offering the degree programme and the municipalities providing the HHC services.

The HHC nurses received a study information sheet from their respective nurse managers and were given an informed consent letter to fill in, providing agreement to participate in the study. The information sheet contained information about the aim of the study, quality improvement process, data collection procedures, informants’ right to self‐determination and aspects of confidentiality. The HHC nurses also received information about who to contact for additional study information. All of those given the study information sheet agreed to participate in the study.

The course teacher for the healthcare administration course, two members of the study research team and the HHC nurses’ nurse manager together created a schedule for the observations. The observation sheet was pilot‐tested when one volunteer HHC nurse was followed and observed by the course teacher during a workday. Following this pilot test, the observation sheet was revised, because the original, precise separation of nursing tasks was seen to be problematic. During the pilot test, for example, it was observed that an HHC nurse could provide emotional support (through a caring conversation) while simultaneously taking care of wounds or be interrupted by a telephone call while dispensing medication.

The course teacher and the HHC nurses’ nurse manager together paired the students and HHC nurses. The participating HHC nurses were guaranteed anonymity, and all information relating to their identity was coded in the data analysis and removed from the research report.

While the students did not participate in any nursing activities, the HHC patients were nonetheless asked whether they consented to the students’ presence during an HHC visit (informed consent).

The observation sheets were collected in separate boxes. All data were collected and are safely stored in a locked filing cabinet at the university where the researchers are employed. The data will not be used for any other purposes other than what was agreed upon with the informants prior to data collection.

## Results

Altogether, 18 HHC nurses were observed during 2–3 work shifts per nurse, and the observation data consisted of 196 observation sheets. The HHC nurses’ background data are presented in Table 2. However, due to their right to anonymity and integrity, their educational level is not presented in detail. In Finland, a specific HHC nursing programme does not currently exist.

**Table 2:** Nurses’ background data (n = 18)

Educational level, freq. (%)	Year of graduation, freq. (%)	HHC work experience, in years, freq. (%)
RN, 8 (44%) RN + specialisation/s, 10 (56%)	1985–1990, 1 (5%) 1991–1996, 1 (5%) 1997–2002, 6 (34%) 2003–2008, 3 (17%) 2009–2014, 6 (34%) 2015–2017, 1 (5%)	<1 year, 3 (17%) 1–3 years, 4 (22.5%) 4–6 years, 2 (11%) 7–9 years, 2 (11%) 10–12 years, 5 (28.5%) 13–15 years, – (0%) 16–18 years, 1 (5%) 19–21 years, 1 (5%)

All of the HHC nurses had a professional nursing education, and the majority (56%) also had one or more specialisations, such as public health nurse, midwife, internal medicine and surgery, or acute care. The HHC nurses received their nursing degrees between 1987 and 2017, with the majority (85%) receiving degrees between 1997 and 2014. Their HHC work experience varied from 1.5 months to 21 years. Due to small subsample sizes, it was not possible to analyse any correlations between the demographic data and other variables.

The observation material consisted of one observation sheet per home visit, and patients can be visited several times during the data collection phase. The majority of HHC patients seen here (Table 3) were female (60%), lived alone (60%), and faced cardiological (47%), endocrinological (13%), oncological (12%) or psychiatric (7%) health problems or a combination of these, or dementia (18%). The majority had different health issues and received care related to medication (57%), blood samples (23%), wound care (17%) or blood pressure (14%). There could be several issues underlying each home visit, and the students documented these as best they could, based mainly on their observations and discussions with the HHC nurses.

**Table 3:** Patients’ background data (n = 196 observation sheets)

Gender, freq. (%)	Living arrangements, freq. (%)	Main diagnosis, freq. (%) One patient could have several diagnoses	Reason for home visit, freq. (%) One visit could have several underlying reasons
Female, 117 (60%) Male, 81 (40%)	Living alone, 118 (60%) Living with, 70 (36%) Data missing, 10 (4%)	Heart/vein diseases, 92 (47%) Dementia/Alzheimer’s, 36 (18%) Diabetes type II, 26 (13%) Cancer, 23 (12%) Schizophrenia, depression, 14 (7%) Genetic disorder, 14 (7%) Parkinson’s disease, 10 (5%) Asthma or COPD, 8 (4%) Rheumatic disease, 7 (3.5%) Arthrosis, 6 (3%) Pain, 5 (2.5%) Stroke/aphasia, 5 (2.5%) Tuberculosis (susp.), 4 (2%) Renal insufficiency, 3 (1.5%) Other, 7 (3.5%) Data missing, 2 (1%)	Medication, 111 (57%) Blood samples, 45 (23%) Wound care, 33 (17%) RR measurement, 28 (14%) Influenza vaccination^a^, 10 (5%) Patient education about medication, 5 (2.5%) Catheterisation, 5 (2.5%) Acute care, 4 (2%) Ear cleaning, 3 (1.5%) Weight measurement, 3 (1.5%) Taking the patient to an appointment with a physician, 3 (1.5%) Pleura drainage, 2 (1%) Care plan meeting, 2 (1%) Patient education about healthcare services, 2 (1%) PEG maintenance, 1 (0.5%) Home visit by physician, 1 (0.5%) First home visit, 1 (0.5%) Data missing, 1 (0.5%)

^a^ The influenza vaccination was analysed separately from the other medication, because in some cases, it was given to the partner of the actual HHC patient; a nurse’s assistance was needed to inject the privately purchased vaccine.

During eight‐hour shifts, the HHC nurses in this sample (n = 196 observation sheets) spent from 75 to 410 minutes (mean = 241 minutes, mode = 242) on indirect patient contact activities (Table 4), which is 50% of the work shift. About 69 minutes (M) was spent on Planning, 50 minutes (M) on Documentation, 48 minutes (M) on Travel, 20 minutes (M) on Telephone calls, 42 minutes (M) on Professional meetings and 40 minutes (M) on Other activities. Thus, despite evidence of some variation, it would appear that indirect patient contact activities comprise a large part of HHC nurses’ daily work.

**Table 4:** Comparison of indirect and direct patient contact activities per work shift

Indirect patient contact activities Mean = 241 minutes	Direct patient contact activities Mean = 27 minutes x 3–6 visits/shift = 81–162 minutes
Planning, 5–100 minutes (M = 69 minutes, Mo = 67 minutes)	Nursing care, 1–125 minutes (M = 15 minutes, Mo = 10 minutes)
Documentation, 14–170 minutes (M = 50 minutes, Mo = 30 minutes)	Medication, 1–215 minutes (M = 17 minutes, Mo = 10 minutes)
Travel, 6–201 minutes (M = 48 minutes, Mo = 36 minutes)	Caring discussion, 1–40 minutes (M = 10 minutes, Mo = 10 minutes)
Telephone calls, 2–63 minutes (M = 20 min, Mo = 10 minutes)	Other activities, 120–200 minutes (M = 40 minutes, Mo = 23 minutes)
Professional meetings, 5–155 minutes (M = 42 min, Mo = 25 minutes)	
Other activities, 3–189 minutes (M = 40 minutes, Mo = 5 minutes)	

The HHC nurses spent from 2 to 196 minutes (M = 27 minutes, Mo = 10 minutes) per patient per visit on direct patient contact activities. On the majority of observation sheets, the only information recorded was categorised under the main categories (Indirect patient contact activities, Direct patient contact activities), with nothing under the subcategories (e.g. Planning, Nursing care). This, however, was not seen to be a detriment, because the subcategories were mainly used to exemplify the main categories and facilitate the students’ observations. About 15 minutes (M) was spent on Nursing care, 17 minutes (M) on Medication and 10 minutes (M) on Caring discussion (could include patient education). Explicit patient education was offered during only 3.5% of home visit. Information recorded under Other activities included the following: waiting for the patient to come and open the door (3–5 minutes), documentation during home visit (3–15 minutes), further situation analysis (5–10 minutes) and acute care situation (only one observation, 60 minutes). It was observed that the data for direct patient contact activities represent nurses’ time management during one home visit and should therefore be multiplied by the average number of home visits per nurse during a work shift, that is 3–6 home visits (Table 4).

From the data, one sees that the HHC nurses spent 50% of their time on indirect patient contact activities and 38% on direct patient contact activities. Of the direct patient contact activities, the majority were illness‐centred and included a focus on clinical symptoms or medication. Altogether, 12% of activities were characterised as unclear (neither indirect nor direct), possibly because the students only recorded activities under the main categories in the observation sheet. It is also possible that this was related to methodical difficulties; the students may have been uncertain about how to record what they observed, for example if many activities occurred simultaneously. Prior to the start of the observations, the students were instructed to record any activities that they perceived as being unclear under the category Other activities in the observation sheet, along with a brief explanation.

## Discussion

The aim of this study was to describe HHC nurses’ activities and time management from the perspective of master’s‐level nursing students. With reference to Benner’s 26 stages of clinical competence, we saw in the analysis that while half of the nurses were not yet experts, the other half were (>7 years of working experience). Looking at their year of graduation, one sees that the majority of the nurses had begun employment in HHC directly after graduation. One can therefore assume that staff turnover in the two HHC settings seen here was quite low (see also Ref. 18.

The majority of the patients in this sample received home visits because of a long‐term illness (cardiological, endocrinological, oncological or psychiatric illness; dementia) or multimorbidity. Also, the majority of activities underlying the home visits could, to some degree, be linked to long‐term illnesses: medication (57%), blood samples (23%), wound care (17%) or measurement of blood pressure (14%). Only a few observations of acute care activities were seen (acute bleeding, rash; congested urinary or pleura drainage). The sample corresponds well with long‐term illness prevalence statistics in Finland. Consequently, it can be considered relevant and comparable to other HHC populations (see also Ref. 1.

Patient education was observed during only 3.5% of home visits. Still, the nurses may have also given emotional support, engaged in caring conversations, encouraged patients’ physical activity or conducted rehabilitative acts while performing other nursing activities. As noted previously, the simultaneous performance of nursing activities could be problematic with regard to characterisation, and the students were instructed to record any activities considered to be unclear under the Other activities category. Despite 12% of the patient sample having cancer and 2.5% having pain, there were no observations of palliative care as such. Based on the findings, we maintain that HHC is quite illness‐centred (see also Ref. 17 and reliant on a focus on clinical symptoms and medication. The HHC services offered here can be considered to partially contribute to hospital avoidance 1 or the enablement of early discharge. Nevertheless, with respect to the patient sample seen here (60% were older persons living alone), we maintain that to avoid risk, HHC must be reorganised and relevant resource allocation established (see also Ref. 21.

In this sample, about half of each eight‐hour work shift was used on indirect patient contact activities 22, 23: Planning (Mo = 67 minutes/shift), Documentation (Mo = 30 minutes/shift), Travel (Mo = 39 minutes/shift), Telephone calls (Mo = 10 minutes/shift), Professional meetings (Mo = 40 minutes/shift) or Other activities (Mo = 40 minutes/shift). Indirect patient contact activities (about 4 hours per eight‐hour shift) appear to form the basis of HHC nurses’ daily work (see also Ref. 5. Still, because the indirect patient activities were related to care coordination and collaboration, we find that they are nevertheless necessary and facilitate the actual home visits (see also Ref. 9.

In comparison, the time spent on direct patient contact activities 22, 23 seems disproportionately small. Again, some activities might have been conducted simultaneously and therefore not noted in the students’ observations. Also, a number of activities were recorded under Other activities, for example waiting for the patient to open the door, documentation or situation analysis during a home visit. We perceive that such activities limited the actual time that the nurses had for direct patient contact activities. Given this and that nurses could simultaneously perform different activities, it is therefore understandable that no observations of explicit patient counselling, psychosocial care, prevention, physical activity or rehabilitation (see also Ref. 17 were recorded.

We still note the possibility that missed care or care left undone due to unrealistic nurse‐to‐patient ratios, as seen by Aiken et al. in a hospital context 27, could be a contributing factor. Also, the observations were dependent on individual student’s observational accuracy, and the structure of the observation sheet may have guided observations too much.

The aim of periodic or continuous HHC is the provision of holistic medical and nursing care. The patients here had different long‐ or short‐term health problems, and diverse nursing activities were required. As seen in the data, there were a number of activities conducted *for the patient*, to promote continuous intra‐ and interprofessional patient care (see also Ref. 5, but fewer nursing activities conducted *with the patient*. Observation data on person‐centeredness as shared decision‐making and/or in meaningful activities in a personalised environment were not available (see also Refs 12, 13, 14), because the structure of the observation sheet did not accommodate such. Indirect patient contact activities consume a lot of time that could be used for health promotion, rehabilitation or other meaningful activities. Nonetheless, indirect patient contact activities are necessary for patient safety and person‐centred care. HHC staff in Finland are more and more comprised of primary care nurses with no advanced education or training. That is, they have no specific training in, for example, clinical screening, decision‐making, patient advocacy, documentation, evidence‐based care, interprofessional collaboration and communication, or person‐centeredness/integrated care. To promote patient safety, patient‐centred care and work satisfaction, the further education of or integrated care management certification for HHC nurses is recommended (see also Ref. 5. The creation of interprofessional teams that are responsible for HHC patients’ intermediate care under the guidance of advanced nurse practitioners (ANPs) or clinical nurse specialists (CNSs) is also recommended. Both ANPs and CNSs have advanced competence in the holistic assessment of patients’ physical and mental status, and in planning, coordinating, implementing and evaluating patient‐centred evidence‐based care and rehabilitation. We maintain that such changes will also improve resource allocation.

### Strengths and limitations

The accuracy of the students’ observations is related to their individual capacity to objectively and selectively observe, which is a limitation. Also, the structure of the observation sheet, which was based on a differentiation between activities involving indirect and direct patient contact 22, 23, may have been challenging or guided the observations too much. For purely economic reasons, we did not include the use of a validated instrument; classification systems are licensed and not freely available. In the future, we recommend that the background data collected with regard to main diagnosis be replaced with a nursing care category. Also, a validated instrument such as the Clinical Care Classification System (CCCS) should be used as a basis for structuring the observation sheet, and case load intensity measurement tools should be integrated into the study design and/or interviews to further investigate nurses’ time management. Still, the students here had received training in observation technique and with regard to the specific observation sheet used here. The observation sheet had also been pilot‐tested and revised prior to data collection. The course teacher’s instruction, which could be considered a form of coaching, might have affected the stringency of the students’ observations positively – or not. To overcome the observer effect, the data were derived from different students’ various observations of multiple HHC nurses over the course of several work shifts, which should raise the study’s inner validity. The findings cannot be generalised to describe HHC in all municipalities in Finland, because the data collection occurred during a certain period of time and the setting was comprised of two municipal HHC organisations.

## Conclusions

Older and/or multimorbid clientele require integrated care skills, which at a minimum necessitates the further education and training of HHC staff or even standardised certification. To ensure integrated, person‐centred, safe patient care, these vital reforms should be immediately implemented alongside the reorganisation of HHC nurses’ work duties, that is HHC structures and processes. Performance effectiveness is related to the training and quality of individual nurses and the way care is organised.

In the observation data, 50% of the care provided by nurses during each eight‐hour work shift was spent on indirect patient contact activities and only 38% on direct patient contact activities. While the indirect patient contact activities did promote continuous intra‐ and interprofessional patient care and patient safety, such a difference could weaken person‐centeredness and rehabilitation. The appropriate balance between indirect and direct patient contact activities should be discussed intra‐ and interprofessionally, delineated and made explicit in nurses’ work plans and nursing documentation, alongside discussions pertaining to relevant resource allocation.

## Impact Statement

What does this paper contribute to the wider global clinical community?

- During an eight‐hour work shift in an HHC setting, nurses spend more time on indirect than direct patient contact activities.
- Health issues related to long‐term illnesses underlie the majority of HHC visits. Thus, one can conclude that HHC is at present quite illness‐centred, with a focus on clinical symptoms and medication instead of person‐centred care.
- To ensure person‐centred, safe patient care in an HHC setting, vital reforms should be immediately implemented. These include standardised certification policies and the delineation of the balance between indirect and direct patient contact activities in nurses’ work plans and nursing documentation, in parallel with relevant resource allocation.

## Conflict of interest

The authors have no conflicts of interests.

## Author contributions

LF was responsible for the pilot study design and the observation sheet structure, and has participated the manuscript writing with critical appraisal. YN was responsible for the leadership course during which the data collection was organised, tested the observation sheet, instructed the master's students in use of it, and participated the manuscript writing. HVR was responsible for the data analysis and had responsibility over the manuscript, submission and correspondence.

## Ethical approval

We have followed the journal’s authorship policy in the Editorial Policies and Ethical Considerations section on eligibility for author listing.

## Funding

This manuscript is part of a research project funded by Eschnerska Foundation, Turku, Finland.
